# A Parallel Phenotypic Versus Target-Based Screening Strategy for RNA-Dependent RNA Polymerase Inhibitors of the Influenza A Virus

**DOI:** 10.3390/v11090826

**Published:** 2019-09-05

**Authors:** Xiujuan Zhao, Yanyan Wang, Qinghua Cui, Ping Li, Lin Wang, Zinuo Chen, Lijun Rong, Ruikun Du

**Affiliations:** 1 College of Pharmacy, Shandong University of Traditional Chinese Medicine, Jinan 250355, China (X.Z.) (Y.Z.) (Q.C.) (P.L.) (L.W.) (Z.C.); 2 Shandong Provincial Collaborative Innovation Center for Antiviral Traditional Chinese Medicine, Jinan 250355, China; 3 Qingdao Academy of Chinese Medicinal Sciences, Shandong University of Traditional Chinese Medicine, Qingdao 266122, China; 4 Department of Microbiology and Immunology, College of Medicine, University of Illinois at Chicago, Chicago, IL 60612, USA

**Keywords:** Influenza A virus, phenotypic screen, target-based screen, RNA dependent RNA polymerase inhibitor

## Abstract

Influenza A virus infections cause significant morbidity and mortality, and novel antivirals are urgently needed. Influenza RNA-dependent RNA polymerase (RdRp) activity has been acknowledged as a promising target for novel antivirals. In this study, a phenotypic versus target-based screening strategy was established to identify the influenza A virus inhibitors targeting the virus RNA transcription/replication steps by sequentially using an RdRp-targeted screen and a replication-competent reporter virus-based approach using the same compounds. To demonstrate the utility of this approach, a pilot screen of a library of 891 compounds derived from natural products was carried out. Quality control analysis indicates that the primary screen was robust for identification of influenza A virus inhibitors targeting RdRp activity. Finally, two hit candidates were identified, and one was validated as a putative RdRp inhibitor. This strategy can greatly reduce the number of false positives and improve the accuracy and efficacy of primary screening, thereby providing a powerful tool for antiviral discovery.

## 1. Introduction

Influenza is an acute respiratory and highly infectious disease that is mainly caused by influenza A viruses (IAVs), posing a serious public health problem globally. According to the World Health Organization (WHO), influenza epidemics are estimated to result in about three to five million cases of severe illness and up to 650,000 respiratory deaths annually [1]. Further, the increase of zoonotic transmission with avian influenza viruses is a growing concern [2,3]. Two classes of anti-influenza drugs are currently available in most countries, including viral ion channel M2 blockers (amantadine and rimantadine) and neuraminidase inhibitors (NAIs; oseltamivir, zanamivir, peramivir, and laninamivir) [4,5]. However, M2 inhibitors are not recommended for clinical use due to widespread resistance, and there is also a concern for NAIs since global circulation of the oseltamivir-resistant seasonal A(H1N1) virus occurred in 2008–2009 [6,7,8]. Numerous drugs with alternate modes of action are in development, including those targeting viral hemagglutinin and RNA-dependent RNA polymerase (RdRp), as well as host components (reviewed in [9,10]).

The influenza virus RdRp is a heterotrimer (comprised of PA, PB2, and PB1), which plays an essential role in viral transcription and replication. The major role attributed to PA during influenza virus infection is the endonuclease activity upon “cap-snatching” for viral transcription initiation in the nucleus of a host cell [11,12]. PB2 contains the cap-binding domain, which recognizes the capped structure on nascent host mRNAs to be cleaved by PA [13,14,15]. PB1 is the RNA polymerizing subunit of the RNA dependent RNA polymerase [16]. It is thought that the RdRp activity is a promising target for novel antivirals, and recent progress in understanding the structure and functions of influenza polymerase complex has facilitated the identification of inhibitors targeting either individual component of the complex or subunits interactions [17,18,19,20,21,22,23,24,25,26,27,28,29]. Among these RdRp inhibitors, Baloxavir Marboxil has been licensed in 2018 in Japan and the US, while Favipiravir is licensed for use in humans in Japan [30,31,32]. 

Different screening approaches, based on cells expressing the influenza virus RdRp complex, nucleoprotein (NP) and a viral mini-genomic RNA, have been recently developed for identifying inhibitors targeting influenza A viral RNA transcription/replication [33,34]. However, these target-based screen approaches often have a high rate of false positives. In this study, we developed a parallel phenotypic versus target-based screen to improve the accuracy and efficacy of screen for influenza A virus RdRp inhibitors, and we demonstrated that this strategy is a powerful approach for inhibitor identification.

## 2. Materials and Methods 

### 2.1. Cell Lines and Viruses

Human embryonic kidney cell line 293T and Madin-Darby canine kidney (MDCK) epithelial cells were obtained from Dr. Fei Deng (Wuhan Institute of Virology, CAS, China) and grown in Dulbecco’s modified Eagle’s medium (DMEM; Cellgro, Manassas, VA, USA) supplemented with 10% fetal bovine serum (FBS; Gibco, Carlsbad, CA, USA), 1000 units/mL penicillin, and 100 μg/mL of streptomycin (Invitrogen, Carlsbad, CA, USA). The influenza A reporter PR8-Gluc virus and wild-type PR8 virus were generated and stocked in our lab as previously described [35].

### 2.2. Compound Library and Control 

The library containing 891 natural products was purchased from MedChemExpress (MCE; Monmouth Junction, NJ, USA). The compounds were arrayed in twelve 96-well plates (columns 2 to 11 on each plate) at a 10 mM concentration in dimethyl sulfoxide (DMSO), leaving columns 1, and 12 with DMSO. The positive control drug for this assay, baloxavir acid (MCE, Monmouth Junction, NJ, USA), was solubilized at 10 mM in DMSO. The stock solution was diluted to a final concentration of 20 μM for the screens.

### 2.3. Cell-Based Influenza RdRp Assay 

The RdRp assay was modified as previously described [35]. Briefly, 293T cells were co-transfected with plasmids for the expression of viral polymerase complex proteins (i.e., PB2, PB1, PA, and NP) and a firefly luciferase gene-encoding influenza viral minigenome using Lipofectamine 2000 (Invitrogen), according to the manufacturer’s instructions. After incubation for 4 h (hrs), the cells were washed with PBS and re-suspended with fresh DMEM.

For assay optimization, the transfected cells were seeded into white, flat-bottom, 96-well plates (CulturPlate; PerkinElmer, Waltham, MA) at a density of 20,000 cells/well in a 100 µL assay medium. Cells expressing PB1, PA, NP, and viral minigenome, but no PB2, were used as the blank control. At 24 h, 48 h, and 72 h post-transfection (p.t.), respectively, 50 µL of neolite luciferase substrate (PerkinElmer) was added to each well, and the plates were incubated at room temperature for 5 to 10 min. Luciferase activity was measured by a Synergy microplate reader (BioTek, Winooski, VT, USA).

To perform HTS, the transfected cells were seeded into white, flat-bottom, 96-well CulturPlates (PerkinElmer) at a density of 20,000 cells/well in an 80 µL assay medium, followed by the addition of each compound in a 20 µL assay medium per well, resulting in a final concentration of 20 µM (0.2% DMSO) for all compounds. In each 96-well plate, DMSO and baloxavir acid were used as negative and positive controls, respectively. Plates were incubated at 37 °C for 24 h, followed by luciferase activity measurement.

For dose-response analysis, 293T cells were co-transfected with plasmids expressing PB2, PB1, PA, NP, and the firefly luciferase gene-encoding influenza viral minigenome. After 4 h of incubation, the cells were washed with PBS, re-suspended with fresh DMEM, and seeded into white, flat-bottom, 96-well CulturPlate (PerkinElmer) at a density of 20,000 cells/well in 80 µL assay medium, followed by adding each compound in 20 µL assay medium per well, resulting in final concentrations of 20–2.5 µM (0.2% DMSO) for each compound by 2-fold dilutions. After 24 h of incubation, the firefly luciferase expressions were determined as described above.

### 2.4. Reporter Influenza Virus (PR8-Gluc) Infection Assay

MDCK cells were seeded into white, flat-bottom, 96-well CulturPlates (PerkinElmer) at a density of 10,000 cells/well and cultured at 37 °C overnight. The next day, the cells were washed with PBS twice, followed by inoculation of the PR8-Gluc virus. Infections were performed in Opti-MEM containing 2 µg/mL N-tosyl-L-phenylalanine chloromethyl ketone (TPCK)–trypsin (Sigma-Aldrich, St. Louis, MO, USA).

The dynamic signal range of the Gluc reporter assay was assessed by infecting MDCK cells with varying quantities of the PR8-Gluc virus (multiplicities of infection [MOIs] of 0.001, 0.01, 0.1, and 1) and determining Gluc activity at various times post-infection (24 and 36 hr p.i.). The Gluc assay was performed using a Pierce *Guassia* luciferase glow assay kit (Thermo scientific, Rockford, IL, USA) according to the manufacturer’s instructions. Mock infected cells were used as a blank control.

To perform HTS, MDCK cells were infected with the PR8-Gluc virus at 0.01 MOI, in the presence of test compounds of 20 µM (0.2% DMSO). In each 96-well plate, DMSO and baloxavir acid were used as negative and positive controls, respectively. Plates were incubated at 37 °C for 36 h, followed by luciferase activity measurement.

### 2.5. Cell Viability

293T cells and MDCK cells were seeded into white, flat-bottom, 96-well CulturPlates (PerkinElmer) respectively at densities of 20,000 and 10,000 cells/well, respectively. For toxicity screening, cells were treated with indicated compounds at 20 μM, while for determination of CC_50_s, cells were treated with increasing concentrations of test compounds.

Cell viability was assessed by using the ATPlite 1step cell viability assay kit (PerkinElmer), according to the manufacturer’s instructions. Briefly, a volume of ATPlite reagent equal to that of the culture media was added to cells in each well. Plates were shaken on a plate shaker for two min to induce cell lysis, incubated at room temperature for 10 min, and subjected to luminescence measurement.

### 2.6. Titer Reduction Assay

Monolayers of MDCK cells grown in 24-well plates were infected with the influenza A PR8 virus at an MOI of 0.01. After 2 h of incubation, Opti-MEM containing 2 µg/mL of TPCK-trypsin as well as various concentrations of JL-5001 or JL-5002 were added. DMSO and baloxavir acid were used as negative and positive controls, respectively. The plates were incubated for 24 h at 37 °C, and supernatants were harvested for virus titration.

### 2.7. Statistical Analysis

The quality of each screen was assessed by evaluating the signal-to-background (S/B) ratio, the coefficient of variation (CV), and the Z’ factors. In each plate, the parameters were calculated as follows: (1) S/B = mean signal of negative control / mean signal of positive control; (2) CV = SD of negative control / mean of negative control; (3) Z’ = 1 − 3 × (SD of positive control + SD of negative control) / (mean of negative control - mean of positive control). SD represents the standard deviation. A Z’ value between 0.5 and 1.0 is considered robust enough for an HTS assay, while CV reflects signal deviation within an assay and is recommended to be less than or equal to 20% [36].

The percent inhibition of the tested compounds was calculated with the following equation: percent inhibition = (signal of negative control − signal of tested compound) / (signal of negative control − signal of positive control) × 100%.

## 3. Results

### 3.1. Establishment of an Influenza a Virus RdRp-Targeted HTS Assay

A cell-based RdRp assay was adapted for high-throughput screening (HTS) to identify inhibitors targeting IAV RNA transcription/replication. Briefly, plasmids expressing IAV NP, PA, PB2, PB1, and a mini-genomic RNA were co-transfected into 293T cells. In constructing the mini-genomic plasmid, the open reading frame of the influenza A/WSN/33 NP protein was replaced by firefly luciferase, and this RNA segment was inserted into a human RNA polymerase I promoter/terminator cassette in the reverse orientation and complementary sense. Transfected cells were re-suspended and seeded into 96-well plates followed by incubation and luciferase measurement.

**Figure 1:**
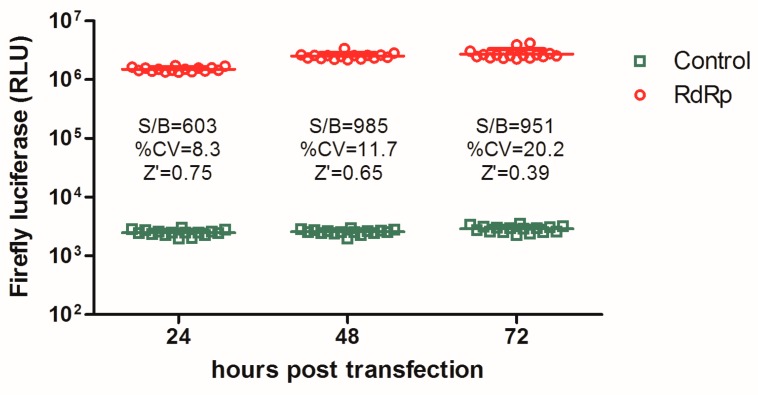
Optimization of cell-based influenza RNA-dependent RNA polymerase (RdRp) assay in a high-throughput screen system. 293T cells transiently expressing viral RdRp proteins (PB2, PB1, PA) and NP, together with a minigenome RNA carrying the firefly luciferase gene, were seeded into 96-well plates (16 replicates) and subjected to a luciferase assay at the indicated times post transfection. The signal-to-background (S/B) ratios, coefficient of variations (CVs), as well as the Z’ factors for each batch of data, were calculated.

In optimizing the screening assay, the luciferase signal was measured at 24 h, 48 h, and 72 h p.t. respectively, and the accuracy was assessed using several statistical parameters, including the S/B ratio, CV, and Z’ factor. As shown in Figure 1, the transfected cells at 24 h p.t. and thereafter produced a robust S/B ratio of >600, which is sufficient for high-throughput screening assays. However, the CV and Z’ value varied among the three batches of data, and, at 24 h p.t., the Z’ value of the assay reached 0.75 with a CV 8.3%, which together meet the desirable requirements for HTS.

### 3.2. Evaluation of a Replication-Competent Reporter Influenza PR8-Gluc Virus for HTS

**Figure 2:**
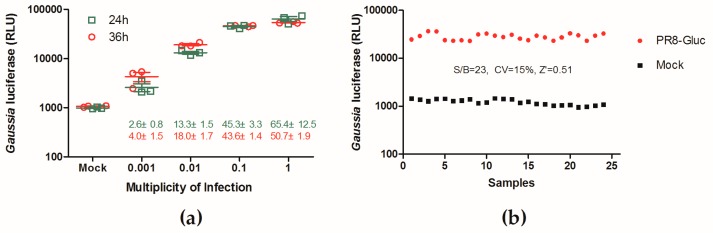
Establishment of a high-throughput screen system using the replication-competent recombinant influenza virus carrying *Gaussia* luciferase, PR8-Gluc. (**a**) Signal range of the PR8-Gluc virus-based infection assay. Madin-Darby canine kidney (MDCK) cells were infected with the reporter influenza virus PR8-Gluc virus at multiplicities of infection (MOIs) of 0.001, 0.01, 0.1, and 1, respectively. Gluc activities were measured at 24 and 36 hr post-infection (p.i.), and those of the mock-infected cells were regarded as background. Data are shown as the average ± standard deviation (*n* = 3). (**b**) Quality control of the PR8-Gluc virus-based screening system. MDCK cells growing in 96-well plates (24 replicates) were infected with a PR8-Gluc virus at an MOI of 0.01, at 36 hr p.i. Gluc activities were measured and the S/B ratio, CV, and Z’ factor were calculated. Mock infected cells were measured for Gluc activities as background.

A phenotypic screening assay was developed using a replication-competent recombinant influenza A virus expressing the *Gaussia* luciferase (Gluc), PR8-Gluc [37]. To determine the dynamic range of the Gluc signal in the reporter assay, various MOIs were used to infect the MDCK cells, and luciferase signals of the infected cells were measured at 24 h and 36 h p.i.. As shown in Figure 2a, upon infection with an MOI of 0.01 (tested at 36 h p.i.), this assay produced an S/B ratio of 18.0 ± 1.7, which is comparable to that of the other HTS assays reported [38]. Although infection at MOIs of 0.1 or 1 could achieve higher S/B ratios (43.6 to 65.4), we observed that infection with higher MOIs often led to less sensitivity in inhibitor identification. Therefore, infection at an MOI of 0.01 for 36 h was chosen for HTS development.

The quality of this HTS assay was further assessed, and the S/B ratio, CV, and Z’ factor were 23, 15%, and 0.51, respectively (Figure 2b), demonstrating that this PR8-Gluc virus-based assay is suitable for HTS.

### 3.3. Development of a Parallel HTS Assay

False positives in the primary screen are one of the major problems with HTS, while strategies with two or more related screens in parallel can greatly reduce the numbers of false positives [39]. Thus, we decided to develop a parallel screening strategy by combining the aforementioned influenza A virus RdRp-targeted assay and the PR8-Gluc virus-based assay for HTS.

**Figure 3:**
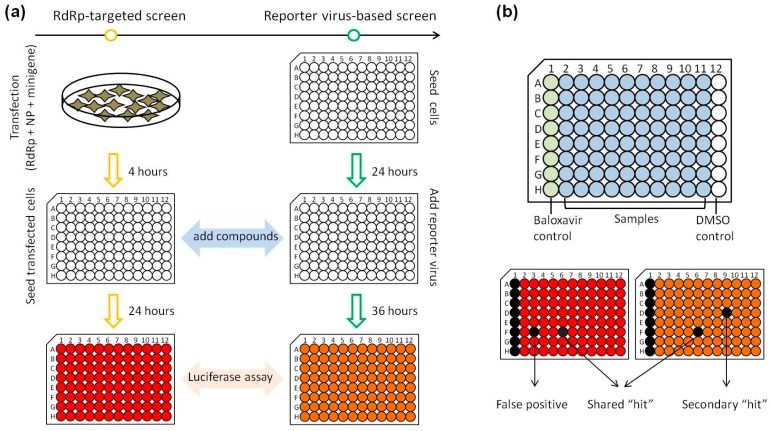
Experimental design of the parallel high-throughput screening strategy. (**a**) RdRp-targeted and PR8-Gluc virus-based screen were sequentially or simultaneously carried out to evaluate the same library of compounds, and the data of each screen were collected for comparison. (**b**) Upper panel: For each plate in both screens, columns 1 and 12 were treated with baloxavir acid and DMSO as positive and negative controls, respectively. Lower panel: A compound that shows an inhibition of >80% in an assay plate is selected as hits (black). Hits shared by two screens are selected as putative RdRp inhibitors; hits specific to PR8-Gluc virus-based screen are secondary hits and not discussed in this study, while hits specific to RdRp-targeted screen are regarded as false positives.

As outlined in Figure 3a, the RdRp-targeted assay and the PR8-Gluc virus-based assay were used to evaluate the same library of compounds sequentially, and the effect of each compound on RdRp activity and influenza A virus infectivity was directly compared. Three classes of hits were identified: (1) hits specific to RdRp activity but not to influenza replication (putative “false positives”); (2) hits shared by both screens were potential RdRp inhibitors; and (3) hits that inhibit influenza A virus infectivity but not RdRp activity (“Secondary hits”; Figure 3b). These secondary hits may potentially target other steps rather than RNA replication/transcription in the influenza virus replication cycle. Of note, cytotoxic compounds and luciferase inhibitors might also be initially identified as shared “hits”, where “toxic hits” can be excluded by a toxicity screening, while an orthogonal counterscreen using wild-type IAVs can exclude the small molecules that inhibit luciferase activity [40].

### 3.4. Pilot Screen of a Compound Library of Natural Products

A pilot screen of a library of 891 compounds derived from the natural products was carried out to validate the parallel screening strategy. Compounds were tested at a final concentration of 20 μM using the RdRp-based assay and the PR8-Gluc virus-based assay, as well as a cytotoxicity assay [40].

**Figure 4:**
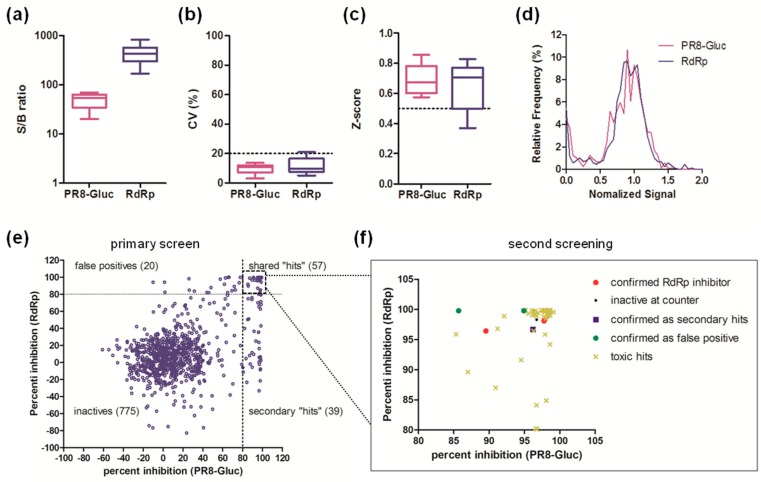
Pilot screen using the parallel high-throughput screen approach. A pilot screen was carried out with a library containing 891 natural products. The quality of primary screen was controlled by assessing the (**a**) S/B ratios, (**b**) CVs, and (**c**) Z’ factors of each plate. The range of the three parameters for each screen were presented by box-and-whisker plots. (**d**) The signal distribution of all 891 samples. (**e**) Primary hit identification strategy. The percent inhibition of each test compound against PR8-Gluc infection was plotted with that against RdRp activity. Compounds showing >80% inhibition in both screens (shared “hits”) are shortlisted for a second screening. (**f**) Second screening. Shared hits identified by the primary screen were subjected to a second round of the parallel screen as well as a cytotoxicity screen. Toxic hits indicate the compounds that reduce cell viability by >30%.

As quality controls, the S/B ratio, CV, and Z’ factor of each plate from both screens were assessed. The S/B ratios ranged from 20.1 to 69.3 for the PR8-Gluc virus-based screen, and 169.2 to 837.1 for the RdRp-targeted screen (Figure 4a). Most CVs were below 20% (Figure 4b), while most Z’ factors were valued above 0.5 (Figure 4c). These data indicate that the overall quality of the pilot screen was excellent.

The normalized signal distribution of each individual screen was bell-shaped, with the majority of the 891 compounds showing no significant effect on the luminescence signal (Figure 4d). As shown in Figure 4e, the percent inhibition of each compound against RdRp activity was plotted against PR8-Gluc virus infectivity, and compounds exhibiting >80% inhibition in both screen (57 shared “hits” in total) were considered as hit candidates and short-listed for second screening. It is interesting to note that the primary screen also identified 20 false positives specific to RdRp activity and 39 secondary hits specific to PR8-Gluc virus infectivity.

Among the 57 “hits” subjected to the second screening, 50 were identified as cytotoxic hits, while five failed to inhibit either RdRp activity or IAV infectivity or both. Nevertheless, two compounds, JL-5001 and JL-5002, were identified to possess putative activity against influenza A virus infection by targeting RdRp (Figure 4f, Table S1). Thus, the screen generates a hit rate of approximately 0.22%.

### 3.5. Identification of JL-5001 as an RdRp Inhibitor

**Figure 5:**
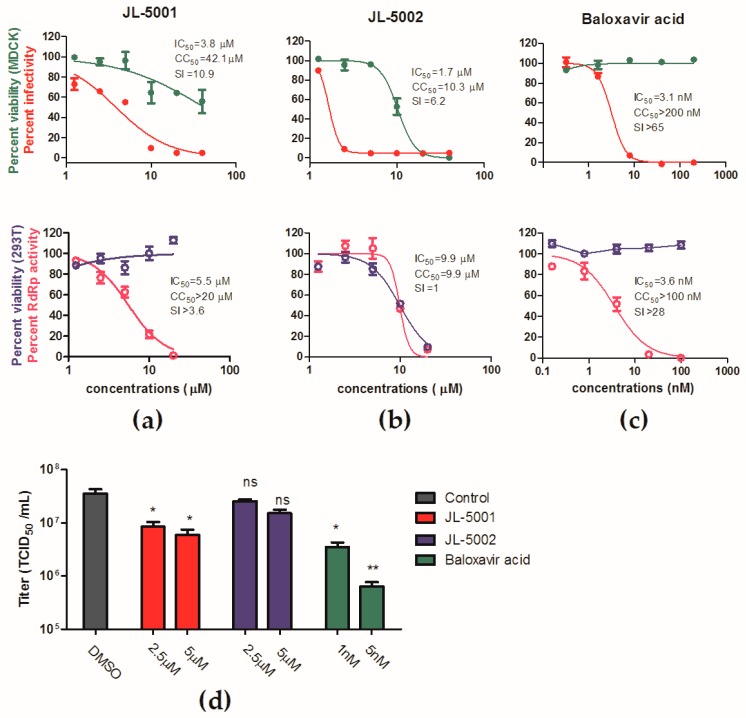
Antiviral determination of JL-5001 and JL-5002. (**a**–**c**) Dose-response curves of (**a**) JL-5001, (**b**) JL-5002, and (**c**) baloxavir acid upon PR8-Gluc virus replication, RdRp activity, as well as viabilities of MDCK and 293T cell lines. The inhibitory effects were analyzed using GraphPad Prism 5. (**d**) MDCK cells were infected with wild-type PR8 virus at an MOI of 0.01 and incubated with indicated concentrations of JL-5001, JL-5002 or baloxavir acid. At 36 hr p.i., the virus titers were determined. ns, no significance; *, *p* < 0.05; **, *p* < 0.01; student’s t test.

To validate the antiviral activity of JL-5001 and JL-5002, a dose-response analysis was performed as previously described [41]. As a result, JL-5001 showed an inhibitory effect against the influenza PR8-Gluc virus with an IC_50_ of 3.8 μM, while the CC_50_ of JL-5001 against MDCK cells was 42.1 μM, corresponding to a SI of 10.9 (Figure 5a). Moreover, the in vitro cell-based influenza RdRp assay demonstrated that JL-5001 inhibited IAV RdRp activity in a dose-dependent manner, with an IC_50_ of 5.5 μM, which is comparable to that against PR8-Gluc virus (Figure 5a). JL-5002 exhibited an IC_50_ of 1.7 μM against the influenza PR8-Gluc virus and a CC_50_ of 10.3 μM against MDCK cells, corresponding to an SI of 6.2 (Figure 2a), however, it showed no significant inhibitory effect on viral RdRp activity (Figure 5b). As a positive control, baloxavir acid was determined to have an IC_50_s of 3.1 nM and 3.6 nM against PR8-Gluc virus and RdRp activity respectively (Figure 5c). 

The effects of JL-5001 and JL-5002 on the in vitro replication of the wild-type IAV PR8 strain were further assessed, in order to exclude the possibility that JL-501 and JL-5002 were luciferase inhibitors. As Figure 5d shows, both JL-5001 and baloxavir acid significantly inhibited virus replication, while JL-5002 reduced virus replication by only twofold at 5 μM (*p* < 0.05, no significance). 

Taken together, these data demonstrate that JL-5001 is a putative IAV inhibitor targeting RdRp activity, while JL-5002 is not a good hit candidate and will not be considered for further development.

## 4. Discussion 

Phenotypic screens and target-based approaches have been the two major screening paradigms for novel drug discovery in the past several decades. Target-based drug discovery has been limited by the small number of well-validated targets. In addition, leads identified through target-based screening often have off-target effects, contributing towards drug toxicity or inactivity in clinical trials [42]. In contrast, phenotypic assays are not limited to known targets, therebu greatly expanding drug target diversity and benefiting the drug discovery process. However, a drug discovery can be hampered due to the unknown modes of action of new lead compounds—for example, the unknown nature of the target made it difficult for lead compound optimization. Therefore, it is desirable to rapidly identify the targets of lead compounds developed through phenotypic screening [43].

For the influenza A virus, either phenotypic screens and target-based approaches are routinely employed for identification of novel antivirals against viral infections [22,24,33,34,38]. Traditionally, phenotypic screens usually use cytopathic effects (CPE) protection as the major assay, while recently replication-competent reporter IAVs have been developed to assess virus replication, providing simple and robust approaches for HTS campaigns [44,45]. A representative target-based screen for IAV inhibitors is RdRp assay using cells transiently or stably expressing influenza vRNP components: four viral proteins (PB2, PB1, PA, and NP) together with a virus-like RNA [33,34]. Although these target (RdRp)-based screens are convenient and reliable, there are several potential pitfalls. First, the reconstituted vRNP may not perfectly reflect the nature of the infection process. Second, hits based on these screening systems may interfere with the PolI transcription machinery. Therefore, some hit candidates may fail when tested using infectious IAVs.

In this study we report a parallel HTS strategy that combines a target (RdRp)-based screen and a phenotypic screen (using reporter influenza A PR8-Gluc virus). The RdRp-based and PR8-Gluc virus-based screening are carried out sequentially upon the same compound library, and only the hits selected by both screens are selected for further development. This strategy can avoid the pitfalls of the target (RdRp)-based screen, by significantly reducing the number of false-positives, and improve the accuracy and efficacy of the primary screen. Moreover, the target of the lead compounds is known as RdRp, and the process of lead compound optimization can be greatly facilitated.

By using this parallel HTS strategy, a library consisting of 891 compounds derived from natural products was screened as a proof-of-concept HTS. Quality controls showed that the primary screen was robust and the data reliable (Figure 4a–d). As a result, 57 “hits” shared by both screens were subjected to a second screening, where 55 of them failed at this stage. The dose-response analysis and titer reduction assay using wild-type IAV further validated JL-5001, one of the two hit candidates, as a potential IAV RdRp inhibitor, thereby providing a novel chemical scaffold for further optimization.

It is interesting to point out that the hits specific to the PR8-Gluc virus-based screen, but not to the RdRp-targeted screen, likely target steps other than RNA transcription/replication in the influenza A virus replication cycle—steps such as virus entry, assembly, and release. These hits will be further studied in the near future.

In summary, our study reports a simple and robust parallel HTS strategy by performing a target (RdRp)-based and a phenotypic (by employing a reporter influenza virus) screen sequentially, to identify influenza inhibitors targeting virus RNA transcription/replication. A pilot screen of a compound library from natural products has identified JL-5001 as a candidate antiviral targeting RdRp. Our work demonstrates that this strategy is a powerful approach to identify influenza A virus inhibitors targeting RdRp and other steps in viral replication. 

## Supplementary Materials

The following are available online at https://www.mdpi.com/1999-4915/11/9/826/s1, Table S1. Results of second screening.
