# Supplementary material for: A Parallel Phenotypic Versus Target-Based Screening Strategy for RNA-Dependent RNA Polymerase Inhibitors of the Influenza A Virus
Source: Viruses. 2019 Sep 5;11(9):826. doi: 10.3390/v11090826 (PMC6783926; doi:10.3390/v11090826)
Supplement: Supplementary file 1 [file viruses-11-00826-s001.pdf]

Supplementary data

S1 Results of second screening

57 hit compounds identified during primary screen were submitted to a second screening. As shown in Table S1, compounds showed cytotoxicity to both MDCK and 293T cells were excluded as toxic hits, while others were identified according to the criteria of primary screen. In summary, 50 toxic hits, 2 inactives, 2 false positive hits, 1 secondary hit and 2 putative RdRp inhibitors were confirmed (Table S1).

Table S1 Results of second screening

No.	compounds	IAV infectivity	viability (MDCK)	RdRp activity	viability (293T)	
**1**	Corosolic acid	0.00	0.89	1.06	0.66	secondary hit
**2**	Doxorubicin (hydrochloride)	0.00	0.55	0.01	0.51	toxic
**3**	Mitomycin C	0.23	0.36	0.09	0.68	toxic
**4**	Parthenolide	0.31	0.78	0.24	0.54	inactive
**5**	Gossypol (acetic acid)	0.00	0.43	0.59	0.59	toxic
**6**	Alisol B (23-acetate)	0.01	0.48	0.88	0.66	toxic
**7**	alpha-Mangostin	0.00	0.00	0.31	0.49	toxic
**8**	Ellipticine (hydrochloride)	0.00	0.64	0.00	0.64	toxic
**9**	Trichostatin A	0.10	0.01	0.15	0.51	toxic
**10**	Isoalantolactone	0.01	0.69	0.05	0.73	hit
**11**	Lasalocid	0.00	0.40	0.02	0.40	toxic
**12**	Lasalocid (sodium)	0.01	0.23	0.01	0.38	toxic
**13**	Chelerythrine Chloride	0.00	0.04	0.00	0.03	toxic
**14**	Nanchangmycin	0.00	0.20	0.00	0.33	toxic
**15**	Dioscin	0.00	0.06	0.00	0.01	toxic
**16**	Cucurbitacin E	0.00	0.61	0.00	0.40	toxic
**17**	Cinobufagin	0.01	0.26	0.00	0.38	toxic
**18**	Digoxin	0.00	0.22	0.01	0.39	toxic
**19**	Homoharringtonine	0.00	0.05	0.00	0.79	false positive
**20**	Cycloheximide	0.01	0.33	0.00	0.55	toxic
**21**	Lycorine (hydrochloride)	0.05	0.49	0.02	0.61	toxic
**22**	Cucurbitacin B	0.15	0.46	0.00	0.51	toxic
**23**	Pristimerin	0.00	0.00	0.00	0.00	toxic
**24**	Gambogic Acid	0.95	0.43	1.20	0.70	inactive
**25**	Lanatoside C	0.00	0.16	0.01	0.35	toxic
**26**	Solamargine	0.00	0.00	0.00	0.00	toxic
**27**	Alantolactone	0.00	0.29	0.00	0.68	toxic
**28**	Triptonide	0.00	0.20	0.00	0.78	false positive
**29**	(S)-10-Hydroxycamptothecin	0.15	0.16	0.06	0.67	toxic
**30**	beta-Mangostin	0.02	0.45	0.78	0.47	toxic
**31**	Saikosaponin D	0.00	0.01	0.39	0.03	toxic
**32**	Bufalin	0.01	0.30	0.01	0.32	toxic
**33**	(-)-Securinine	0.16	0.41	0.00	0.55	toxic
**34**	Timosaponin AIII	0.01	0.87	0.09	0.90	hit
**35**	Bufotalin	0.00	0.15	0.01	0.42	toxic
**36**	Shogaol	0.00	0.28	0.00	0.05	toxic
**37**	Tubercidin	0.00	0.01	0.00	0.01	toxic
**38**	Resibufogenin	0.00	0.43	0.01	0.26	toxic
**39**	Tripterin	0.00	0.12	0.00	0.09	toxic

**40**	Puromycin (Dihydrochloride)	0.01	0.03	0.00	0.57	toxic
**41**	Salinomycin	0.01	0.57	0.19	0.68	toxic
**42**	Daunorubicin (Hydrochloride)	0.00	0.16	0.00	0.63	toxic
**43**	Sanguinarine (chloride)	0.00	0.00	0.00	0.01	toxic
**44**	Tubeimoside I	0.01	0.01	0.71	0.30	toxic
**45**	Halofuginone	0.00	0.00	0.00	0.57	toxic
**46**	Cinobufotalin	0.00	0.21	0.01	0.38	toxic
**47**	Tanshinone I	0.00	0.01	0.09	0.27	toxic
**48**	Oridonin	0.00	0.02	0.00	0.07	toxic
**49**	Ailanthone	0.00	0.42	0.00	0.32	toxic
**50**	Arenobufagin	0.00	0.06	0.00	0.56	toxic
**51**	Digitoxin	0.01	0.46	0.01	0.42	toxic
**52**	Dehydrocostus Lactone	0.00	0.00	0.00	0.01	toxic
**53**	Gamabufotalin	0.00	0.19	0.01	0.39	toxic
**54**	Dihydroisotanshinone I	0.00	0.00	0.00	0.67	toxic
**55**	Digitonin	0.00	0.00	0.01	0.02	toxic
**56**	Iberin	0.14	0.27	0.04	0.69	toxic
**57**	Morusin	0.00	0.04	0.14	0.47	toxic
**PC**	baloxavir acid 1	0.02	0.92	0.00	1.07	
	baloxavir acid 2	0.02	1.01	0.00	1.04	
	baloxavir acid 3	0.02	0.99	0.00	1.04	
